# Research Progress on Elesclomol-Induced Cuproptosis for Antitumor Effects

**DOI:** 10.3390/biomedicines14040910

**Published:** 2026-04-16

**Authors:** Lingzhi Peng, Na Sun, Biqiong Ren

**Affiliations:** School of Integrated Chinese and Western Medicine, Hunan University of Chinese Medicine, Changsha 410208, China; 20242132@stu.hnucm.edu.cn (L.P.); sunna@ncst.edu.cn (N.S.)

**Keywords:** elesclomol, cuproptosis, cancer treatment, immune microenvironment, drug resistance mechanisms, combination therapy, nanodelivery systems

## Abstract

Cuproptosis represents a novel form of programmed cell death that relies on copper ions and targets the mitochondrial tricarboxylic acid cycle, offering fresh avenues for tumor therapy. Elesclomol, as a highly efficient small-molecule copper ion carrier, transports copper ions into mitochondria. Under the action of ferredoxin-1 (FDX1), it induces abnormal aggregation of lipoylated proteins and loss of iron–sulphur clusters, thereby generating protein toxicity stress and killing tumor cells. Furthermore, elesclomol effectively remodels the tumor immune microenvironment by promoting dendritic cell maturation and CD8^+^ T cell infiltration, demonstrating synergistic effects with immune checkpoint blockade therapies. However, tumor cells can develop resistance mechanisms through metabolic reprogramming via hypoxia-inducible factor-1α (HIF-1α) and the nuclear factor E2-related factor 2 (Nrf2)-driven reductive pathway, which partially limits the drug’s clinical efficacy. Addressing this limitation, combination therapies integrating elesclomol with targeted agents such as ferroptosis inducers or chemotherapeutic drugs have demonstrated significant antitumor advantages. Future research must urgently leverage the selection of precise biomarkers and the development of novel intelligent nanodelivery systems to further advance the safe and efficient clinical translation of elesclomol.

## 1. Introduction

**Figure 1:**
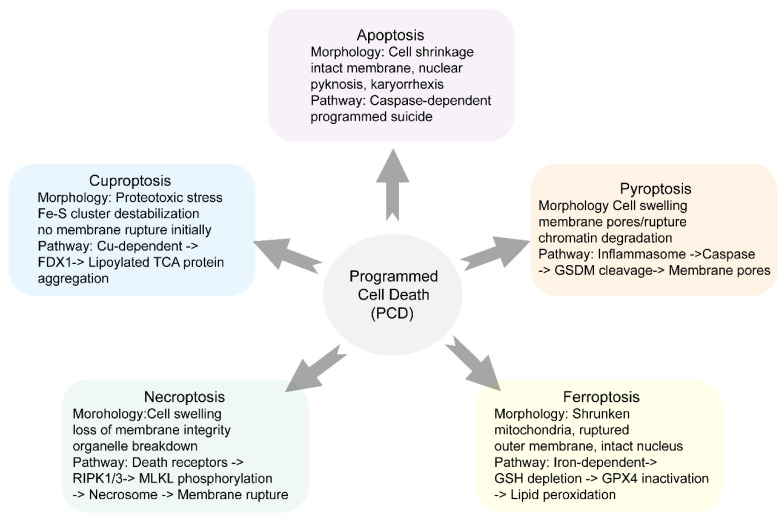
Schematic diagram of PCD subtypes and their key morphological and molecular characteristics. Apoptosis manifests as cell shrinkage and nuclear chromatin condensation; pyroptosis mediates membrane pore formation via inflammasomes and GSDM; ferroptosis depends on iron and lipid peroxidation; necroptosis involves the RIPK1/3-MLKL pathway leading to membrane rupture; copper death induces protein toxicity stress via copper-dependent TCA protein aggregation.

Cuproptosis was formally defined in 2022 as a novel, regulated form of cell death [1]. Unlike apoptosis, pyroptosis, ferroptosis, and other cell death pathways, cuproptosis is not primarily driven by excessive accumulation of reactive oxygen species (ROS). Instead, it is triggered by abnormal intracellular copper ion accumulation directly targeting key proteins undergoing lipoylation modification within the mitochondrial tricarboxylic acid cycle (TCA). Its molecular hallmarks include: abnormal binding of copper ions to acylated proteins inducing their aggregation, loss of iron–sulphur (Fe-S) clusters, impaired mitochondrial respiration, and consequent protein toxicity stress, ultimately leading to cell death [2]. The proposal of copper death not only enriches the theoretical framework of regulated cell death but also redefines copper’s biological role in cellular life processes, offering novel research directions for therapeutic strategies targeting tumor metabolic vulnerabilities. Schematic representations of programmed cell death (PCD) subtypes and their key morphological and molecular characteristics are illustrated in Figure 1.

Under normal physiological conditions, intracellular copper homeostasis is subject to precise regulation; however, copper metabolism imbalance is prevalent in numerous malignant tumors and is closely associated with tumor proliferation, angiogenesis, invasion, metastasis, and treatment resistance. Previous studies indicate that moderately elevated intracellular copper levels can activate pro-tumor signaling pathways such as hypoxia-inducible factor-1α (HIF-1α), PI3K/AKT, and MAPK, thereby promoting tumor progression. However, when copper ions accumulate excessively within cells and exceed their buffering and homeostasis maintenance capacity, pronounced cytotoxic effects occur [3,4]. This suggests copper exhibits a “double-edged sword” characteristic in tumor biology, with its biological effects highly dependent on intracellular copper ion concentration, valence state, and subcellular distribution.

In 2019, two research teams led by Golub and Peter Tsvetkov jointly discovered a small-molecule compound, elesclomol (ES), capable of binding to copper ions (Cu^2+^) and transporting it into tumor cells. However, at the time, this phenomenon was not subject to in-depth interpretation [5]. ES is a small-molecule copper ion carrier initially developed as a mitochondrial oxidative stress inducer for antitumor therapy [6]. The molecular core of elesclomol comprises a malonic acid moiety, with an N-methyl-phenylthiocarbamoyl hydrazide group attached to each end. This structure, owing to its thio-carbonyl (C=S) group, efficiently chelates divalent Cu^2+^, forming a stable 1:1 stoichiometric complex (ES-Cu). This complex exhibits strong lipophilicity and redox activity, enabling preferential entry into and enrichment within mitochondria. This triggers mitochondrial metabolic disruption and protein homeostasis imbalance, ultimately inducing tumor cell death [7,8]. Although early clinical and basic research suggested Isimomib’s antitumor potential, its precise molecular mechanism of action remained unclear for a considerable period.

This paper aims to provide a systematic review of the molecular mechanisms by which elesclomol induces cuproptosis, focusing on its dual role in tumor cell killing and immune microenvironment remodeling. It further analyses resistance mechanisms mediated by hypoxia and redox homeostasis. Additionally, the paper discusses research advances in combination therapy strategies and novel delivery systems for elesclomol, with the objective of providing theoretical foundations for the clinical translation and optimized application of copper-dependent cell death-targeted therapies.

## 2. Literature Search Strategy

The literature included in this review was retrieved from PubMed and Web of Science. Articles published in English up to February 2026 were considered. Keywords used for retrieval included “cuproptosis”, “elesclomol”, “ferroptosis”, “regulated cell death”, “mitochondrial metabolism”, “copper metabolism”, “copper complexes”, “Disulfiram”, “mitochondrial metabolism”, “TCA cycle”, “lipoylation”, “FDX1”, “DLAT”, “Fe-S cluster”, “proteotoxic stress”, “apoptosis”, “necroptosis”, “pyroptosis”, “cancer” and “cancer therapy”. Priority was given to original research articles and high-impact reviews published in the past five years, while seminal earlier studies were also included where relevant.

## 3. Copper Homeostasis Imbalance-Driven Tumor Cell Death

### 3.1. The Regulatory Network of Intracellular Copper Homeostasis and Apoptotic Signaling

Appropriate intracellular concentrations of Cu^2+^ can activate a series of downstream signaling pathways, a phenomenon particularly prevalent in tumor cells. However, the relationship between Cu^2+^ and tumors remains insufficiently understood at present. Cu^2+^ can influence the activation of the Notch signaling pathway. It has been reported that Cu^2+^ promotes the shedding of Jagged-1 protein (the ligand for Notch molecules) from the surface of tumor cells and facilitates tumor cell metastasis [9]. However, the binding of Notch/Jagged-1 on the cell membrane surface promotes tumor cell proliferation [10]. Therefore, the specific molecular mechanisms by which Cu^2+^ regulates the Notch signaling pathway require further investigation.

Cu^2+^ can increase the expression of hypoxia-inducible factor-1α (HIF-1α) in tumor cells and inhibit its ubiquitin-mediated degradation pathway within the cells [11]. HIF-1α is one of the key factors promoting the progression of various tumors, including lung cancer, glioma, and liver cancer [12,13,14]. Therefore, a certain concentration of Cu^2+^ within tumor cells can exert a tumor-promoting effect. Moreover, intracellular Cu^2+^ can activate multiple signaling pathways associated with cell growth, including the Akt pathway [15], ERK pathway [16], and JNK pathway [17]. The primary mechanism by which elesclomol induces tumor cell death lies in its ability to continuously transport Cu^2+^ into tumor cells, leading to intracellular copper overload and thereby triggering cytotoxic effects.

### 3.2. Mechanism of Elesclomol-Induced Cuproptosis

Cuproptosis requires the entry of Cu^2+^ into cells to be triggered. Elesclomol and disulfiram are two classic compounds that facilitate the transfer of Cu^2+^ into cells. Disulfiram was the first drug discovered to facilitate Cu^2+^ uptake into cells. Researchers observed that diethyl dithiocarbamate, a metabolite of disulfiram in vivo, chelates Cu^2+^. Through a series of mechanisms, this prevents effective degradation of intracellular proteins, leading to cell death due to protein overload. This mechanism has been identified as a potential approach for antitumor therapy [18].

Elesclomol, as a classic inhibitor of the mitochondrial oxidative respiratory chain, is currently the most commonly used compound for inducing cuproptosis. Wu et al. utilized mass spectrometry and X-ray diffraction techniques to elucidate the process by which erythromycin carries Cu^2+^ into cells [19]. At a molar ratio of 1:1, elesclomol and Cu^2+^ form a novel compound—elesclomol copper (ES-Cu)—which enters the cellular mitochondria. Here, under the influence of FDX1, the Cu^2+^ within the ES-Cu complex is reduced to Cu^+^, thereby losing its capacity to bind with elesclomol. The Cu^2+^-depleted elesclomol rapidly returns to the extracellular space, rebinds with Cu^2+^, and re-enters the mitochondria to repeat the cycle. This ultimately leads to intracellular Cu^2+^ accumulation, inducing oxidative stress that causes cell death.

**Figure 2:**
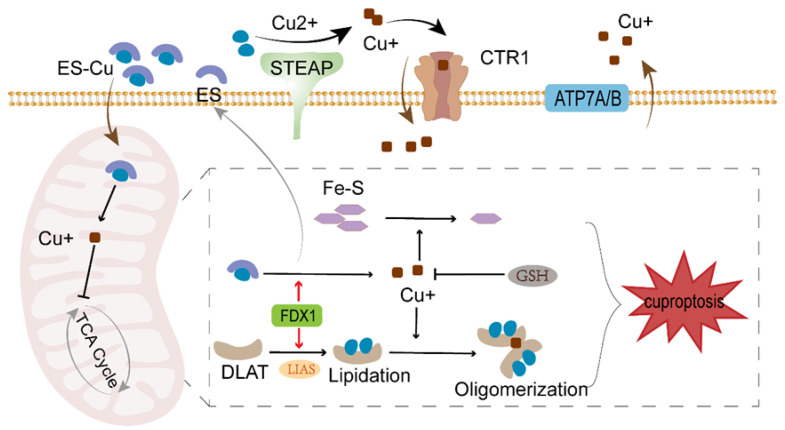
Schematic representation of the molecular mechanism by which elesclomol induces cuproptosis. ES binds with Cu^2+^ to form a complex that enters the mitochondria. Under the action of FDX1, Cu^2+^ is reduced to toxic Cu^+^. Subsequent accumulation of Cu^+^ leads to abnormal oligomerization of acylated proteins such as DLAT, concurrently causing loss of Fe-S cluster proteins. This ultimately triggers protein toxicity stress, resulting in cell death.

Of particular note is that FDX1 not only reduces Cu^2+^ to Cu^+^ within mitochondria but also cooperates with lipoic acid synthase (LIAS) to regulate the lipoylation of multiple proteins [20,21]. Following the reduction of Cu^2+^ by FDX1, it can immediately acylate the cysteine residues of proteins such as DLAT, glycine cleaving system protein H (GCSH), dihydrotioctic acid succinyl transferase (DLST), and dihydrotioctic acid branched-chain transferase E2 (DBT) [22,23,24]. Proteins undergoing lipidation may form abnormal aggregates upon binding with Cu^+^ ions. For instance, when Cu^+^ binds to DLAT, the resulting aggregated DLAT is unable to mediate the conversion of pyruvate—the end product of glycolysis—into acetyl-CoA. This subsequently inhibits the TCA cycle [25]. Furthermore, Fe-S cluster proteins are extensively depleted during cellular cuproptosis, leading to impaired electron transport in oxidative respiration and reduced cellular activity [26]. A schematic diagram illustrating the mechanism by which elesclomol induces cuproptosis is shown in Figure 2.

## 4. The Role of Elesclomol-Induced Cuproptosis in Cancer Therapy

Since Peter Tsvetkov’s team conducted a comprehensive and in-depth analysis of the pharmacological mechanism of elesclomol, research into its antitumor properties has gradually gained momentum. The key to eliciting its antitumor effects lies in the targeted delivery of elesclomol chelated with Cu^2+^ (ES-Cu) into tumor cells. Given that elesclomol lacks inherent targeting capabilities, the design of nanomedicine particles represents a crucial future direction for its clinical application. Guo et al. reported a nanotechnology approach [17] wherein ES-Cu was encapsulated within a reactive oxygen species-responsive nanomaterial to form NP@ESCu. Experiments revealed that upon entering tumor cells, NP@ESCu not only induces cuproptosis but also remodels the tumor microenvironment. This promotes the maturation of dendritic cells from an immature to a mature phenotype, enhances CD8^+^ T cell infiltration within tumor tissue, and induces the polarization of tumor-associated macrophages (TAMs) from the M2 to the M1 phenotype. Consequently, this activates the antitumor immune response. Moreover, they discovered that in animal models of bladder cancer, the combination of NP@ESCu with anti-PD-L1 antibodies significantly enhanced in vivo antitumor efficacy. However, this effect was virtually absent when elesclomol failed to bind Cu^2+^. This finding demonstrates that elesclomol possesses potent potential antitumor activity whilst also regulating the tumor microenvironment in vivo, offering crucial guidance for future novel antitumor therapies.

The activation or blockade of immune checkpoints constitutes a highly significant strategy in antitumor immunotherapy. The cuproptosis response process can reshape the tumor microenvironment and exert synergistic effects with immune checkpoint inhibitors. Current research predominantly focuses on the PD-1/PD-L1 pathway, though other pathways, such as CTLA-4 and LAG-3, have also been implicated [27]. Overall, cuproptosis inducers such as elesclomol reshape the immune microenvironment, thereby prompting tumor cells to produce substantial quantities of tumor-associated antigens (TAAs) and damage-associated molecular patterns (DAMPs), including HMGB1, ATP, calreticulin, and others [28,29]. These injury signals can strongly activate dendritic cells, promoting their maturation and migration to lymph nodes. This enables more effective presentation of tumor antigens to T cells, thereby initiating a tumor-specific T cell response [30]. Furthermore, drugs such as elesclomol can internalize Cu^2+^ from the tumor microenvironment. The substantial presence of extracellular Cu^2+^ in the microenvironment induces cuproptosis in tumor-infiltrating T cells, leading to functional exhaustion. Internalizing Cu^2+^ alleviates the exhausting effects of free Cu^2+^ on T cells [31]. At this juncture, the combined use of anti-PD-L1 or anti-CTLA-4 antibodies can synergistically enhance the antitumor effect.

**Figure 3:**
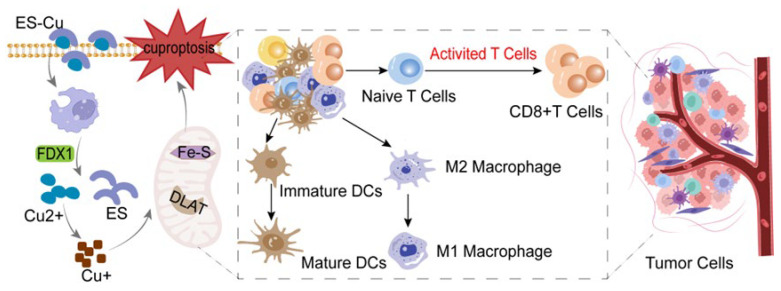
Elesclomol induces cuproptosis in tumor cells to enhance immune responses. Following elesclomol-induced cuproptosis in tumor cells, the release of damage-associated molecular patterns (DAMPs) and tumor antigens promotes dendritic cell (DC) maturation and M1-type macrophage polarization. This subsequently activates CD8^+^ T cell infiltration and tumor cell killing, thereby reshaping the immunosuppressive microenvironment.

Similarly, Lu et al.’s research indicates that the nanomedicine ES@CuO, constructed from elesclomol and copper oxide nanoparticles, has also achieved breakthrough progress in animal experiments for tumor treatment [32]. Through investigations in a melanoma animal model, they discovered that upon entering melanoma cells, CuO within the weakly acidic tumor environment generated Cu^2+^, triggering cuproptosis in the tumor cells. Concurrently, the ES@CuO nanomedicine increased the number of tumor-infiltrating lymphocytes and the secretion of inflammatory cytokines, thereby reshaping the immunosuppressive tumor microenvironment. When ES@CuO was combined with an anti-PD-1 antibody for immunotherapy, it further enhanced the efficacy against melanoma. Luo et al. reported the role of a Cu^2+^-based metal–organic framework nanoplatform (Cu-MOF) in antitumor immunity [33]. They encapsulated elesclomol within Cu-MOF to form ES-Cu-MOF nanomaterials, discovering that these materials significantly triggered cell death within tumor cells while enhancing dendritic cell maturation and cytotoxic CD8^+^ T cell infiltration within the tumor microenvironment. These findings demonstrate that ES-Cu-based nanomaterial drugs effectively induce cuproptosis in tumor cells, laying the groundwork for developing novel elesclomol-based tumor treatment strategies. Elesclomol induces cuproptosis in tumor cells to enhance immune responses, as shown in in Figure 3.

## 5. Tumor Resistance Mechanisms and Multiple Challenges

### 5.1. The Tumor Microenvironment and Intrinsic Resistance Mechanisms

#### 5.1.1. Hypoxic Microenvironments Inhibit Cuproptosis in Tumor Cells

**Figure 4:**
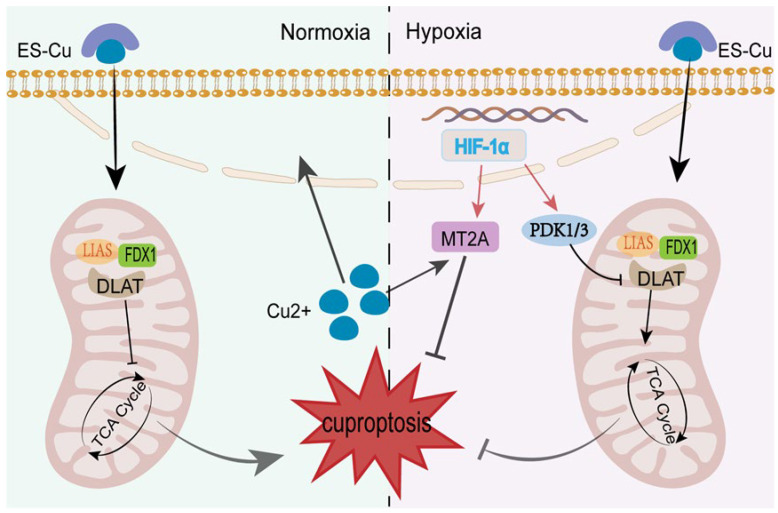
Schematic representation of HIF-1α inhibiting cuproptosis. Within hypoxic microenvironments, the elevated expression of HIF-1α induces resistance through a dual mechanism: Firstly, HIF-1α upregulates PDK1/3, leading to the phosphorylation and ubiquitin-mediated degradation of the key target protein DLAT, thereby reducing the “substrate” for cuproptosis. Concurrently, HIF-1α induces metallothionein MT2A expression, wherein MT2A directly chelates intracellular Cu^2+^, preventing its mitochondrial entry and subsequent toxic reactions, thereby inhibiting cuproptosis.

HIF-1α serves as a pivotal regulator for tumor cell survival under hypoxic conditions, inhibiting cuproptosis by modulating cellular metabolism and metal homeostasis. This is achieved primarily through two pathways: metabolic reprogramming and copper homeostasis regulation. In the former scenario, as DLAT is a key molecule in cellular cuproptosis, the high expression of HIF-1α within tumor cells promotes the expression of pyruvate dehydrogenase kinase 1 and pyruvate dehydrogenase kinase 3 (PDK1/3). These kinases phosphorylate serine residues at the S100 site of DLAT. Subsequently, phosphorylated DLAT is recognized by E3 ubiquitin ligases, leading to ubiquitin-mediated degradation. Reduced DLAT expression directly compromises the stability of the lipoylated protein complex. Cu^2+^ requires targeting lipoylated proteins to induce cell death; when lipoylated protein stability diminishes, leading to degradation, Cu^2+^ cannot exert its cuproptosis-inducing effect. This represents a key mechanism underpinning tumor cell resistance to compounds such as elesclomol [34]. The second crucial mechanism by which HIF-1α resists cuproptosis involves forming stable compounds through Cu^2+^ binding. HIF-1α recognizes and binds to the hypoxia response element on the metallothionein 2A (MT2A) gene promoter, significantly upregulating MT2A expression levels. MT2A robustly binds various divalent metal ions within cells. Upon ESCu entry, MT2A directly binds Cu^2+^, subsequently forming stable complexes. This prevents Cu^2+^ from entering mitochondria and interacting with key cellular molecules such as DLAT, thereby inhibiting oxidative stress induction and blocking the core trigger mechanism of cellular cuproptosis [35]. Tumor tissues such as hepatocellular carcinoma, breast cancer, and colorectal cancer typically exhibit high expression of HIF-1α [36,37,38]. The efficacy of cuproptosis drug treatment in these tumors is significantly compromised. Therefore, pre-treatment with HIF-1α inhibitors to eliminate HIF-1α influence offers a promising new therapeutic strategy for treating refractory solid tumors, particularly copper-rich or hypoxic tumors. A schematic representation of HIF-1α inhibiting cuproptosis is shown in Figure 4.

#### 5.1.2. Feedback-Mediated Upregulation of the Intracellular Reduction System in Tumor Cells Suppresses Cuproptosis

**Figure 5:**
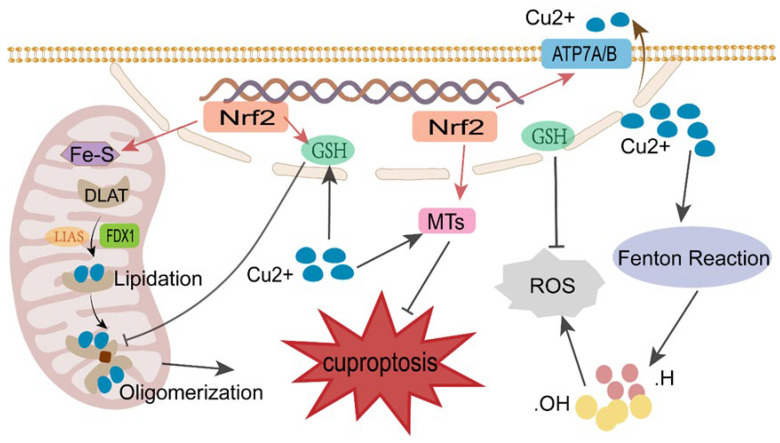
Schematic representation of the Nrf2 reduction system inhibiting cuproptosis. The Nrf2 signaling pathway constructs a defense system through multiple mechanisms, including upregulating ATP7A/B to promote copper ion efflux, inducing metallothionein (MT) and GSH synthesis to neutralize copper toxicity and ROS, and maintaining Fe-S cluster protein homeostasis, thereby protecting cancer cells from cuproptosis damage.

The reducing systems present within tumor cells primarily originate from two sources: reduced glutathione (GSH) and reduced nicotinamide adenine dinucleotide phosphate (NADPH). These reducing agents neutralize intracellular reactive oxygen species (ROS), thereby shielding cells from oxidative stress. The Nrf2 signaling pathway represents the foremost source of reductive products, regulating downstream GSH production. Moreover, the Nrf2/GSH reduction system can inhibit cuproptosis through multiple complex mechanisms, including: (1) Promoting Cu^2+^ efflux, where Nrf2 upregulates the expression of copper transporters ATP7A and ATP7B. These proteins primarily function to pump excess Cu^2+^ out of the cell or sequester it within specific organelles, thereby reducing the concentration of free copper ions in the cytoplasm. This represents the most direct and central mechanism for inhibiting cuproptosis [39]. (2) Induction of metallothionein expression: Similar to HIF-1α, Nrf2 can also induce the expression of multiple cysteine-rich metallothioneins (MTs). The thiol groups within the cysteine residues of these MTs efficiently bind Cu^2+^ to form non-toxic copper complexes. This prevents Cu^2+^ from binding to crucial cuproptosis proteins such as DLAT within mitochondria, thereby blocking the core triggering step of cuproptosis [40]. (3) Promoting GSH synthesis: Nrf2 directly activates the expression levels of genes encoding the glutamate-cysteine ligase catalytic subunit (GCLC) and regulatory subunit (GCLM). These two subunits collectively form glutamate-cysteine ligase (GCL), the rate-limiting enzyme in GSH synthesis. The abundant thiol groups in GSH chelate free Cu^2+^, thereby inhibiting the induction of cuproptosis [41]. (4) Neutralizing ROS: Although cuproptosis does not primarily depend on substantial ROS levels for initiation, Cu^2+^ itself possesses potent oxidative properties. It can catalyze the Fenton reaction, generating ROS that accumulate and damage cells. The Nrf2-driven synthesis of GSH and the antioxidant system effectively scavenge these copper-induced ROS, providing a protective buffering environment that indirectly inhibits cuproptosis [42,43]. (5) Nrf2 regulates the stability of multiple lipidated proteins within the TCA cycle. These lipidated proteins are prone to misfolding under Cu^2+^ induction, leading to the formation of toxic aggregates; Nrf2 may confer resistance to Cu^2+^-induced misfolding [44]. (6) It promotes the expression of Fe-S cluster proteins, which serve as essential cofactors for mitochondrial respiratory chain complexes I, II, and III, as well as certain enzymes in the TCA cycle (e.g., stellate acid synthase) [45,46]. By maintaining Fe-S cluster protein homeostasis, Nrf2 safeguards the integrity of the mitochondrial electron transport chain, thereby supporting cellular survival and repair mechanisms [47]. A schematic representation of the Nrf2 reduction system inhibiting cuproptosis is shown in Figure 5.

### 5.2. Multiple Limitations of Elesclomol

Elesclomol, as a copper ion carrier, demonstrates potential as a novel antitumor drug in the field of cancer therapy. However, its application remains constrained by multiple limitations.

Firstly, from the perspective of clinical efficacy, elesclomol has demonstrated suboptimal antitumor effects in cancer patients. According to published clinical data, the efficacy of elesclomol in cancer patients is relatively limited, whether used alone or in combination with paclitaxel [48]. The most prominent clinical evaluation was the Phase III SYMMETRY trial (NCT00522834) for stage IV metastatic melanoma [49]. Although the trial was prematurely terminated due to an imbalance in overall survival within the high-LDH patient cohort, retrospective stratification revealed that patients with normal baseline LDH levels achieved significant clinical benefit. These clinical findings underscore that whilst elesclomol is a potent cuproptosis inducer, its clinical efficacy remains highly contingent upon the tumor’s metabolic state. This highlights the urgent need for precise biomarkers (such as LDH levels) and rational combination strategies to overcome its current clinical limitations. Table 1 summarizes the various clinical trials of elesclomol in cancer.

Secondly, the mechanism of action of elesclomol requires further elucidation. Although the concept of cuproptosis has been proposed in recent years and has significantly advanced our understanding of elesclomol’s anti-cancer mechanism, the precise molecular mechanisms underlying cuproptosis remain to be thoroughly investigated. How elesclomol specifically interacts with copper ions within cancer cells, how this interaction ultimately leads to cell death, and whether distinct tumor cell types exhibit differing cuproptosis regulatory mechanisms, all warrant further investigation. Concurrently, these uncertainties currently constrain researchers’ ability to optimize and enhance elesclomol more effectively.

Moreover, the potential mechanisms of resistance to the antitumor activity of elesclomol in tumor cells also constitute a significant factor limiting the application of elesclomol. As elesclomol requires binding to its target molecule FDX1 to transport Cu^2+^ into cells, thereby promoting FDX1’s reduction of Cu^2+^ and exerting its antitumor effect, the expression level of FDX1 constitutes the intrinsic molecular mechanism governing elesclomol’s antitumor efficacy. Quan et al. investigated FDX1 in hepatocellular carcinoma and found that its protein expression levels were significantly lower in cancerous liver tissue compared to normal liver tissue. Furthermore, FDX1 expression levels were positively correlated with both the sensitivity of hepatocellular carcinoma patients to oxaliplatin and their prognosis [51]. Further investigations revealed that FDX1 expression in hepatocellular carcinoma cells is regulated by two non-coding RNAs: LINC02362 and hsa-miR-18a-5p. Upregulating LINC02362 enhances FDX1 expression levels, thereby increasing hepatocellular carcinoma cells’ sensitivity to elesclomol and improving the therapeutic efficacy of oxaliplatin. Another study on prostate cancer resistance revealed that whilst enzalutamide demonstrated initial efficacy in treating castration-resistant prostate cancer, resistance rapidly developed. This resistance also suppressed the efficacy of elesclomol. Mechanistic investigations revealed that enzalutamide treatment suppressed FDX1 expression levels in prostate cancer cells. It is hypothesized that this may inhibit intracellular Cu^2+^ reduction reactions, thereby diminishing the antitumor efficacy of elesclomol [52].

Beyond the expression levels of FDX1 being a key factor governing the antitumor efficacy of elesclomol, the intracellular reduction system also represents another significant mechanism for suppressing cuproptosis. Cuproptosis shares similarities with ferroptosis in terminal molecular pathways, including the requirement for substantial reactive oxygen species production to induce cellular oxidative stress. Consequently, intracellular reductive products that inhibit ferroptosis, such as glutathione (GSH) and glutathione peroxidase 4 (GPX4), may serve as potential factors suppressing cuproptosis [53]. Current academic research in this area remains insufficient. In a study examining cisplatin-resistant lung cancer, Medhi et al. discovered that elevated ROS activity within resistant cell lines could induce the cytotoxic effects of elesclomol, whereas normal cells, possessing higher levels of glutathione (GSH), were able to evade the cytotoxic action of elesclomol [54]. Wang et al. also noted in their investigation of the shared pathways between ferroptosis and cuproptosis that elevated GSH expression suppresses intracellular ROS production, conferring resistance to the ferroptosis inducer Erastin [55]. Whilst they did not address whether high GSH levels might impact the antitumor efficacy of elesclomol, analysis of elesclomol’s mechanism of action and the molecular mechanisms of cuproptosis indicates that Cu^2+^ is reduced to Cu^+^ within cells, inevitably inducing substantial production of ROS-like products. At this stage, elevated GSH levels can reduce ROS species such as hydroxyl radicals, thereby preventing their effective cytotoxic effects. Consequently, it can be inferred that the expression levels of intracellular reduction systems and their products represent a significant potential factor constraining the therapeutic efficacy of elesclomol. However, the precise molecular mechanisms underlying this inhibitory effect warrant further investigation and elucidation.

Finally, as an emerging antitumor therapeutic agent, significant gaps remain regarding the patient populations for whom elesclomol is suitable and its combination regimens with other drugs. Firstly, difficulties exist in patient selection. Clinical trial results indicate elesclomol may be more effective in specific patient cohorts, such as those with lower serum LDH levels [56]. However, accurately identifying such patients and elucidating the molecular mechanisms underpinning this enhanced efficacy—specifically, the intrinsic regulatory pathways governing this synergistic effect—remain current challenges. This necessitates further exploration of biomarkers associated with elesclomol sensitivity to enable more precise patient selection.

In summary, elesclomol faces multiple limitations in tumor treatment, including restricted clinical efficacy, an unclear mechanism of action, difficulties in patient selection, and the need for optimization of combination therapy strategies. With ongoing research into its mechanism of action and the progression of clinical trials, it is anticipated that these limitations may be overcome, offering more effective treatment options for cancer patients.

## 6. Combination Therapy Strategies and Prospects for Elesclomol

### 6.1. Combination Regimens with Drugs Such as Ferroptosis Inducers

Several studies have commenced exploring the use of elesclomol in combination therapies for tumors, achieving notable progress. For instance, recent research investigating cuproptosis in human ovarian cancer revealed that anisomycin induces cuproptosis in human ovarian cancer stem cells. Mechanistically, anisomycin reduced levels of reductive substances such as GSH in ovarian cancer stem cells while increasing levels of pyruvate, lipid peroxides (LPO), and malondialdehyde (MDA). Concurrently, anisomycin was found to modulate the expression levels of cuproptosis-associated genes such as FDX1 and DLAT to a certain extent. Consequently, the combination of anisomycin with elesclomol may represent a viable therapeutic approach for human ovarian cancer or other tumors. The study proposed that anisomycin reduces intracellular reducing agents, which simultaneously act as inhibitors of ferroptosis. Thus, the combination of anethole and elesclomol may facilitate the dual induction of cuproptosis and ferroptosis in ovarian cancer cells, delivering a dual strike against these tumors [57]. In investigating the therapeutic efficacy of elesclomol against gastric cancer, Sun et al. discovered that elevated intracellular Cu^2+^ concentrations promote increased expression of methyl transferase-like protein 16 (METTL16). METTL16 methylates the mRNA of FDX1, a key gene in cuproptosis, thereby enhancing FDX1 translation and facilitating elesclomol-mediated cuproptosis in gastric cancer cells. However, the deacetylase SIRT2 within gastric cancer cells can inhibit METTL16 expression by suppressing its acetylation levels, thereby mediating resistance to elesclomol. The combined application of elesclomol and the SIRT2-specific inhibitor AGK2 significantly enhances the therapeutic efficacy against gastric cancer both in vivo and in vitro [58].

Wang et al. detailed findings demonstrating that the ferroptosis inducers sorafenib and erastin suppress cell growth in primary hepatocellular carcinoma cells by enhancing cuproptosis [59]. Experiments revealed that sorafenib and erastin dose-dependently inhibited HCC cell growth by inducing ferroptosis through increased lipid peroxidation. Further investigations demonstrated that both ferroptosis inducers potentiated cuproptosis induced by the copper ion transporter elesclomol, manifested as enhanced aggregation of lipoylated proteins. Mechanistically, sorafenib and Erastin elevated protein lipoylation levels by inhibiting FDX1 degradation mediated by mitochondrial matrix-associated proteases. They concurrently reduced intracellular GSH synthesis by suppressing cystine/glutamate reverse transporters, thereby increasing free Cu^2+^ concentrations. In a nude mouse xenograft model, the combined use of sorafenib with Elesclomol-Cu significantly inhibited the in vivo growth of hepatocellular carcinoma cells, confirming the potency of ferroptosis inducers in enhancing cuproptosis. These findings propose that the combined targeting of ferroptosis and cuproptosis using ferroptosis inducers and copper ion carriers may represent a novel and effective therapeutic strategy for primary liver cancer.

Moreover, the combination of elesclomol with other drugs has demonstrated promising preliminary results in treating various tumors, including colorectal cancer [60], prostate cancer [61], and pancreatic cancer [62]. A thorough elucidation of its pharmacological and molecular biological mechanisms will significantly aid in optimizing future combination therapies and enhancing treatment efficacy.

### 6.2. Future Directions

Future research on elesclomol must first deepen its understanding at the mechanistic level. On the one hand, it is essential to systematically elucidate the cross-regulatory networks between multiple cell death pathways, including cuproptosis and ferroptosis. Particular attention should be paid to the functional differences in core lipoylation-related molecules such as FDX1, DLAT, and LIAS across various tumor types, as well as the impact of hypoxia, acidification, and metabolic reprogramming within the tumor microenvironment on cuproptosis susceptibility. Concurrently, identifying precise biomarkers to predict elesclomol efficacy will be pivotal for clinical translation. Potential candidates include FDX1 expression levels, copper ion concentrations in tumor tissue or serum, lactate dehydrogenase (LDH) levels, and the tumor’s overall redox state. These markers could assess patient sensitivity to elescomol or its combination regimens, enabling patient stratification and personalized treatment strategies that significantly enhance clinical utility. At the drug and technology level, the development of novel cuproptosis inducers and innovations in delivery systems hold equally significant promise [63]. Particularly in the fields of structural modification and drug design, significant potential exists for designing and synthesizing novel copper complex derivatives with enhanced antitumor activity, using elesclomol as the lead compound. For instance, the development of multinuclear metal complexes such as binuclear complexes warrants consideration. These novel derivatives may more efficiently disrupt intracellular redox homeostasis through synergistic catalysis by dual metal centers, demonstrating superior mitochondrial targeting and enhanced ability to induce lipoylated protein aggregation. Building upon this foundation, advancements in artificial intelligence and computational pharmacology enable the application of deep learning models for virtual screening of extensive compound libraries [64]. This approach predicts compounds’ binding affinity to copper ions, mitochondrial targeting potential, and capacity to induce lipoylated protein aggregation, thereby accelerating the discovery of novel copper death inducers or elismod derivatives—such as the aforementioned highly active binuclear copper complexes. Concurrently, developing nanotechnology-based intelligent delivery platforms that respond to multiple tumor microenvironment cues will emerge as a key direction. This includes creating nanocarriers highly sensitive to pH or specific enzymes, enabling precise release of erlotinib, novel copper complexes, or combination therapies at tumor sites. Such approaches reduce systemic toxicity while enhancing local antitumor efficacy. Looking ahead, an integrated strategy combining artificial intelligence-driven drug discovery, novel metal complex design, precise biomarker screening, and intelligent nanodelivery platforms holds promise for advancing elesclomol and its derivative drugs from experimental research towards more mature, safer, and more effective clinical application stages.

## 7. Summary

Elesclomol, as a first-generation cuproptosis inducer and novel oxidative stress inducer, has demonstrated potent antitumor activity with low normal cell toxicity in preclinical models, proving the immense potential application value of cuproptosis in cancer therapy. However, based on existing clinical trial data and complex in vivo resistance mechanisms, erlotinib has not yet been successfully translated into routine clinical use. It is primarily employed as an experimental tool for investigating cuproptosis pathways and antitumor pharmacological mechanisms. The impediments to its clinical translation stem, on the one hand, from the incomplete elucidation of its precise target mechanisms within the body. On the other hand, although emerging nanotechnologies have been reported to significantly enhance the antitumor effects of elesclomol, such nanodelivery systems still face formidable barriers: complex and costly preparation processes, the instability of physicochemical properties at the nanoscale, and insufficient active targeting and recognition capabilities for tumor cells. Therefore, future research on elesclomol should fully leverage its utility as a probe molecule to conduct an in-depth analysis of the intricate molecular regulatory network governing cuproptosis. This will provide a robust theoretical foundation for designing and developing a new generation of cuproptosis-targeting drugs that possess greater clinical translational potential, enhanced safety, and improved efficacy.

## Figures and Tables

**Table 1:** Summary of major clinical trials of elesclomol in cancer therapy.

ClinicalTrials.gov Identifier	Phase	Cancer Type /Condition	Intervention Strategy	Status	Clinical Outcomes	Ref.
NCT00522834	Phase 3	Stage IV Metastatic Melanoma	Elesclomol + Paclitaxel vs. Paclitaxel	Terminated	Trial was stopped early due to an overall survival imbalance favoring the control group in the high-LDH subgroup. Retrospective analysis showed significant clinical benefit in patients with normal baseline LDH.	[49]
NCT00084214	Phase 2	Stage IV Metastatic Melanoma	Elesclomol + Paclitaxel vs. Paclitaxel	Completed	Met its primary endpoint. The combination doubled the median progression-free survival (PFS) compared to paclitaxel alone, which directly led to the Phase 3 SYMMETRY trial.	[50]
NCT00888615	Phase 2	Platinum-Resistant Ovarian, Fallopian Tube, or Primary Peritoneal Cancer	Elesclomol Sodium + Paclitaxel	Completed	The combination was well tolerated but the objective tumor response rate (19.6%) did not meet the prespecified efficacy threshold to warrant further unstratified investigation.	[48]
NCT00087997	Phase 2	Soft-Tissue Sarcomas	Elesclomol + Paclitaxel	Completed	Evaluated the safety and efficacy of elesclomol in enhancing the antitumor response of weekly paclitaxel. Results primarily cataloged in clinical registries.	No relevant references
NCT00827203	Phase 1	Advanced Metastatic Solid Tumors	Elesclomol Sodium	Terminated	Dose-escalation safety study designed to determine the maximum tolerated dose (MTD) and characterize pharmacokinetics. Trial was halted early.	No relevant references

## Data Availability

No new data were created or analyzed in this study. Data sharing is not applicable to this article.

## Abbreviations

The following abbreviations are used in this manuscript:

HIF-1α	Hypoxia-Inducible Factor 1 Alpha
JNK	c-Jun N-Terminal Kinase
AKT	Protein Kinase B
ATP	Adenosine Triphosphate
CTLA-4	Cytotoxic T-Lymphocyte-Associated Protein 4
DCs	Dendritic Cells
GSH	Glutathione
GPX4	Glutathione Peroxidase 4
LDH	Lactate Dehydrogenase
MAPK	Mitogen-Activated Protein Kinase
NADPH	Nicotinamide Adenine Dinucleotide Phosphate (Reduced Form)
PCD	Programmed Cell Death
PD-1	Programmed Death 1
PD-L1	Programmed Death-Ligand 1
ROS	Reactive Oxygen Species
TCA	Tricarboxylic Acid Cycle
PI3K	Phosphoinositide 3-Kinase
Nrf2	Nuclear Factor Erythroid 2-Related Factor 2
ES	Elesclomol
FDX1	Ferredoxin 1
DLAT	Dihydrolipoamide S-Acetyltransferase
DBT	Dihydrolipoamide Branched-Chain Transacylase E2
ES-Cu	Elesclomol–Copper Complex
LIAS	Lipoic Acid Synthase
GCSH	Glycine Cleavage System Protein H
DLST	Dihydrolipoamide S-Succinyltransferase
TAMs	Tumor-Associated Macrophages
TAAs	Tumor-Associated Antigens
DAMPs	Damage-Associated Molecular Patterns
PDK1/3	Pyruvate Dehydrogenase Kinase 1/3
MT2A	Metallothionein 2A
MTs	Metallothioneins
